# Identification of aberrantly expressed glycans in gastric cancer by integrated lectin microarray and mass spectrometric analyses

**DOI:** 10.18632/oncotarget.13539

**Published:** 2016-11-24

**Authors:** Xiang Li, Feng Guan, Dongliang Li, Zengqi Tan, Ganglong Yang, Yanli Wu, Zhaohui Huang

**Affiliations:** ^1^ Wuxi Oncology Institute, Affiliated Hospital of Jiangnan University, Wuxi, Jiangsu, China; ^2^ College of Life Science, Northwest University, Xi’an, Shannxi, China; ^3^ The Key Laboratory of Carbohydrate Chemistry & Biotechnology, Ministry of Education, School of Biotechnology, Jiangnan University, Wuxi, China; ^4^ Bioimaging Core, Faculty of Health Sciences, University of Macau, Taipa, Macau SAR, China

**Keywords:** gastric cancer, lectin microarray, lectin histochemistry, mass spectrometry, N-glycan

## Abstract

Cancer progression is usually associated with alterations of glycan expression patterns. Little is known regarding global glycomics in gastric cancer, the most common type of epithelial cancer. We integrated lectin microarray and mass spectrometry (MS) methods to profile glycan expression in three gastric cancer cell lines (SGC-7901, HGC-27, and MGC-803) and one normal gastric epithelial cell line (GES-1). Significantly altered glycans were confirmed by lectin staining and MALDI-TOF/TOF-MS. The three cancer cell lines showed increased levels of core-fucosylated N-glycans, GalNAcα-Ser/Thr (Tn antigen), and Sia2-6Galβ1-4GlcNAc N-glycans, but reduced levels of biantennary N-glycans, Galβ1-3GalNAcα-Ser/Thr (T antigen), and (GlcNAc)_n_ N-glycans. Lectin histochemistry was used to validate aberrant expression of four representative glycans (core-fucosylation, Sia2-6Galβ1-4GlcNAc, biantennary N-glycans, T antigen, recognized respectively by lectins LCA, SNA, PHA-E+L, and ACA) in clinical gastric cancer samples. Lower binding capacity for ACA was correlated with significantly poorer patient prognosis. Our findings indicate for the first time that glycans recognized by LCA, ACA, and PHA-E+L are aberrantly expressed in gastric cancer, and suggest that ACA is a potential prognostic factor for gastric cancer.

## INTRODUCTION

Gastric cancer is a common epithelial cancer and the second leading cause of cancer death worldwide [1, 2]. In China, ∼400,000 new cases of gastric cancer and ∼300,000 deaths from the disease were reported during 2005-2010, making it the second most common type of cancer [3, 4]. New prognostic biomarkers are needed to facilitate early diagnosis of cancer, improve treatment outcome, and prolong patient survival. Early detection of gastric cancer typically relies on endoscopic examination, and diagnostic accuracy depends on the skill of the endoscopist [5]. More reliable tissue or serum biomarkers for early diagnosis of gastric cancer are highly desirable.

Glycosylation is a common post-translational modification estimated to occur in >70% of human proteins [6]. It plays crucial roles in molecular recognition, cell-cell adhesion, molecular trafficking, receptor activation, signal transduction, and endocytosis [7]. Aberrant protein glycosylation often occurs during malignant transformation, leads to formation of specific tumor-associated glycans, and is associated with tumor invasiveness, metastasis, and overall poor prognosis [8, 9].

Several studies have suggested aberrant expression of glycans in gastric cancer cells. Serum levels of sialyl Lewis^x^, sialyl Lewis^a^, and sialyl-Tn antigens were elevated in gastric cancer patients [10]. Ishizuka et al found that complex type N-glycans were accumulated in gastric cancer cell lines MKN7 and MKN45 [11]. Huang et al [12] proposed that two lectins (VVA and MPL) are potential biomarkers for distinguishing gastric cancer from gastric ulcer. However, to date glycans have not been extensively applied as clinical tumor biomarkers because of (i) technical difficulties in glycan purification and analysis, and (ii) variations in accuracy depending on the analytical method and type of equipment [13]. An integrated strategy is needed for identification of aberrantly expressed glycans in gastric cancer.

In the present study, we used an approach integrating lectin microarray and mass spectrometry (MS) to quantitatively analyze glycan expression in gastric cancer cells and normal gastric epithelial cells, and confirmed our results in clinical gastric cancer tissues and their adjacent noncancerous tissues. Our findings indicate that (i) core-fucosylation, biantennary N-glycans, and Galβ1-3GalNAcα-Ser/Thr (T antigen), recognized respectively by the lectins LCA, PHA-E+L, and ACA, are aberrantly expressed in gastric cancer, and (ii) ACA is a potential prognostic tool for gastric cancer.

## RESULTS

### Glycopattern analysis of normal gastric and gastric cancer cell lines

**Figure 1:**
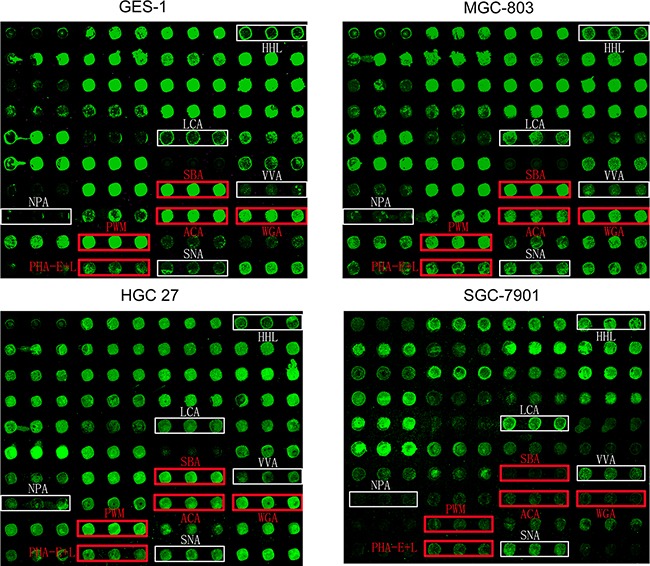
Glycan profiles of gastric cancer and normal gastric cell lines obtained by lectin microarray analysis Differentially expressed glycans in three gastric cancer cell lines (SGC-7901, HGC-27, and MGC-803) and one normal gastric epithelial cell line (GES-1). White boxes: fluorescence intensities of lectins higher in gastric cancer cells. Red boxes: fluorescence intensities of lectins lower in gastric cancer cells. Each sample was observed consistently by three repeated slides. One representative slide was shown.

Patterns of glycoproteins reflect the expression, function, and metabolism of oligosaccharides in cells. We used lectin microarrays containing 37 lectins (Supplementary Table S2), two negative controls (BSA), and one positive control (Cy3-BSA) (Supplementary Figure S1) to analyze fine glycan structures of glycoproteins in GES-1, SGC-7901, HGC-27, and MGC-803 cells. Significant differences (fold change >1.5 observed in at least two gastric cancer cell lines) of glycans recognized by 10 different lectins were observed for cancer cells in comparison with normal cells (Figure 1). Gastric cancer cells had higher expression of five glycan structures recognized by lectins HHL, LCA, NPA, VVA, and SNA, and lower expression of five glycan structures recognized by SBA, PWM, ACA, WGA, and PHA-E+L (Table 1).

**Table 1:** Differential glycopatterns of four cell lines (three gastric cancer and one normal gastric epithelia), determined by lectin microarray analysis

	MGC-803/GES-1	HGC-27/ GES-1	SGC-7901/GES-1		
HHL	1.131	1.707	1.904	Non-substituted α1-6Man	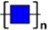
LCA	1.007	1.532	1.915	Fucα1-6GlcNAc (core)	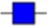
SNA	0.613	1.567	1.839	Sia2-6Galβ1-4GlcNAc	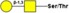
VVA	1.134	1.610	1.583	GalNAcα-Ser/Thr (Tn)	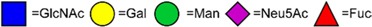
NPA	1.424	1.695	1.099	Non-substituted α1-6Man	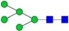
SBA	0.966	0.758	0.445	Terminal GalNAc (especially GalNAcα1-3Gal)	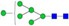
PHA-E+L	1.096	0.610	1.054	Biantennary N-glycans	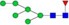
WGA	1.101	0.885	0.699	(GlcNAc)_n_	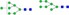
PWM	0.941	0.770	0.616	GlcNAc	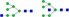
ACA	0.896	0.596	0.730	Galβ1-3GalNAcα-Ser/Thr	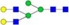

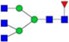

Increased staining by SNA (recognizing Sia2-6Galβ1-4GlcNAc), LCA (recognizing Fucα1-6GlcNAc), VVA (recognizing GalNAcα-Ser/Thr (Tn antigen)) indicated elevated levels of α2-6 sialylation, core-fucosylation, and Tn antigen in MGC-803, HGC-27, and SGC-7901 cells, respectively. Fluorescence intensity of HHL and NPA (recognizing non-substituted α1-3 and α1-6Man) was also higher in these gastric cancer cell lines, suggesting increased expression of high-mannose-type N-glycans. Reduced fluorescence intensity of PHA-E+L and ACA indicated down-regulation of tri-and tetra-antennary structures and Galβ1-3GalNAcα-Ser/Thr structure, and reduced SBA, WGA, and PWM fluorescence intensity indicated lower expression of terminal GalNAc and GlcNAc, in gastric cancer cells (Figure 1B; Table 1).

**Figure 2:**
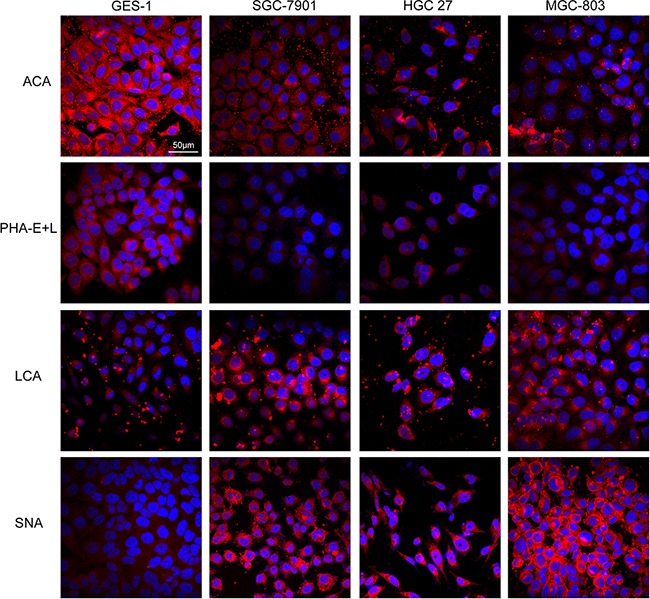
Expression of glycans in the four cell lines evaluated by lectin staining GES-1, SGC-7901, HGC-27, and MGC-803cells were stained with Cy3-labeled lectins (ACA, PHA-E+L, LCA, SNA). Signals in merge images of Cy3-conjugated lectins and DAPI staining of nuclei are shown. Objective magnification: 60x.

Because the glycans recognized by ACA, PHA-E+L, LCA, and SNA are known to be aberrantly expressed in several types of cancer, we selected these four lectins for the validation of differentially expressed glycans in our experimental cell lines. The three gastric cancer cell lines showed higher fluorescence signal intensities of LCA and SNA but lower intensities of PHA-E+L and ACA, consistent with the results of lectin microarray analysis (Figure 2).

### N-Glycan profiles obtained by matrix-assisted laser desorption/ ionization time-of-flight mass spectrometry (MALDI-TOF/TOF-MS)

N-Glycans are involved in cell-cell and cell-matrix interactions, receptor-mediated functions, and specific protein functions [14, 15]. Aberrant N-glycosylation patterns have been observed in many types of tumors, suggesting the potential use of cancer-associated N-glycans as tumor biomarkers [16–18]. We used MALDI-TOF/TOF-MS to profile total N-glycans in GES-1, SGC-7901, HGC-27, and MGC-803 cells and observed abnormal N-glycosylation of gastric cancer.

**Figure 3:**
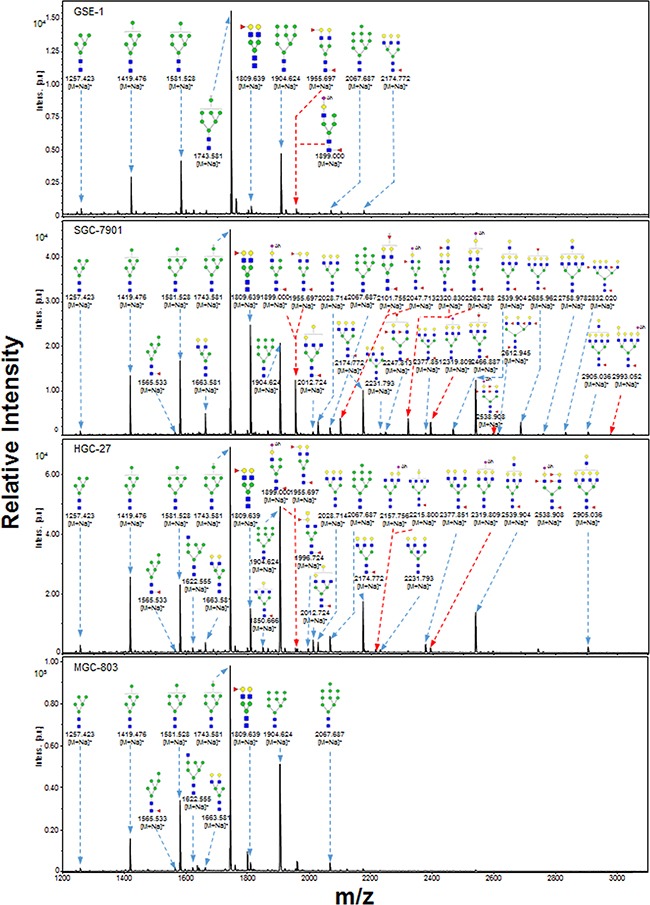
MALDI-TOF/TOF-MS spectra of total N-glycans in the four cell lines N-glycans were characterized in the four cell lines (GES-1, SGC-7901, HGC-27, and MGC-803) by MALDI-TOF/TOF-MS as described in M&M. Representative spectra from triplicate experiments are shown. Detailed glycan structures were analyzed using the GlycoWorkbench program. Proposed structures and their m/z values are shown for each peak.

**Figure 4:**
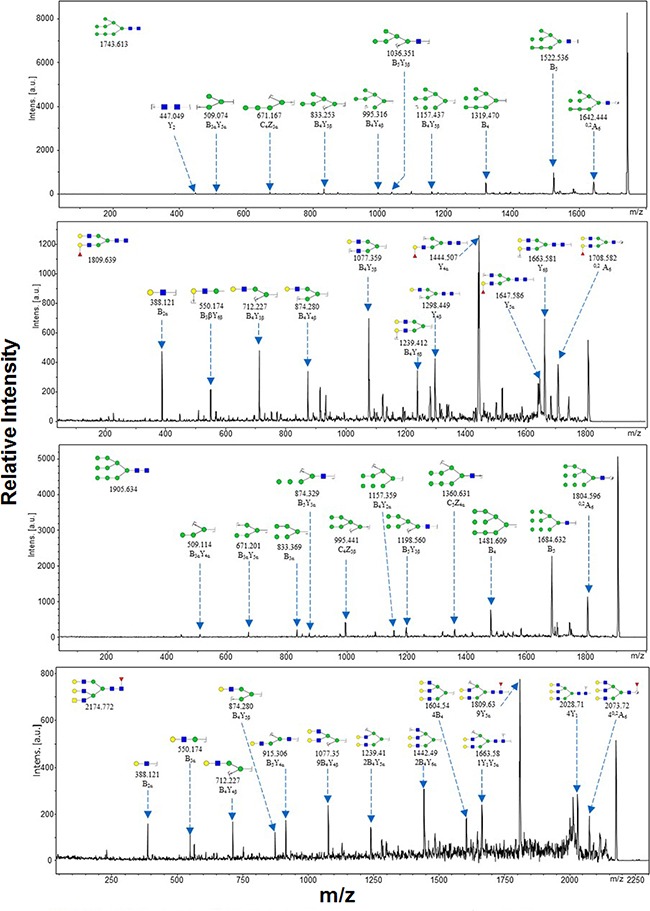
MALDI-TOF/TOF-MS/MS analysis of N-glycan precursor ions in MS spectra of the four cell lines Precursor ions were subjected to MS/MS analysis to obtain cleavages, including B, Y, C, and Z glycosidic cleavages and A and X cross-ring cleavages. Structures of cleavage ions and m/z values are shown in tandem mass spectra. The four major N-glycan peaks are: 1743.613 m/z, 1809.639 m/z, 1905.634 m/z, and 2174.772 m/z.

Representative MALDI-TOF/TOF-MS spectra of N-glycans with signal-to-noise ratios >3 from total glycoproteins were annotated using the GlycoWorkbench software program (Figure 3). GES-1, SGC-7901, HGC-27, and MGC-803 cells showed 9, 22, 18, and 10 distinct m/z N-glycans in both two independent experiments, respectively. Nine N-glycan structures were present in all four cell lines. SGC-7901 and HGC-27 cells had ten and four unique N-glycan structures, respectively (Table 2). MALDI-TOF/TOF-MS analysis *per se* is not sufficient to yield detailed glycan composition. Therefore, MALDI-TOF/TOF-MS was further performed to obtain detailed information regarding substitutions and branching patterns of monosaccharide constituents. Tandem MS spectra of precursor ions with m/z 1743.613, 1809.639, 1905.634, and 2174.772 are shown in Figure 4. Structures of mannose branches were revealed by fragment ions B_4_Y_3β_ (833.253) and B_4_Y_5β_ (1157.437) in m/z 1743.613. The presence of fucose was indicated by B_4_Y_6β_ (1239.41) in m/z 1809.639 and by Y_1_ (2028.714) in m/z 2174.772.

**Table 2:** Proposed structures and their molecular ions in MALDI spectra of N-glycans in the four cell lines

No.	Calculated m/z	Relative intensity				Glycan structure	Calculated m/z (acetyl hydrazine modification)	Glycan structure (sialic)
		GES-1	SGC-7901	HGC-27	MGC-803			
1	1257.423	0.048±0.001	0.024±0.002	0.078±0.045	0.018±0.009	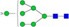	-	-
2	1419.476	0.189±0.001	0.352±0.080	0.294±0.142	0.172±0.008	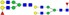	-	-
3	1565.533	-	0.020	0.021	0.026±0.003	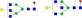	-	-
4	1581.528	0.245±0.035	0.365±0.052	0.307±0.023	0.351±0.008	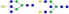	-	-
5	1622.555	-	-	0.021	0.025±0.018	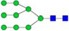	-	-
6	1663.581	-	0.104±0.011	0.048	0.023±0.014	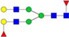	-	-
7	1688.613			0.082±0.025		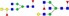		
8	1743.581	1.000	1.000	1.000	1.000	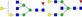	-	-
9	1809.639	0.059±0.022	0.624±0.046	0.199±0.044	0.043±0.0004	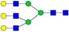	-	-
10	1850.666	-	-	0.054±0.041	-	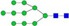	-	-
11	1865.654	-	0.076±0.074	0.039±0.030	-	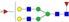	-	-
12	1905.634	0.382±0.106	0.438±0.066	0.724±0.050	0.476±0.003	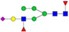	-	-
13	1955.697	0.048±0.025	0.294±0.142	0.020	-	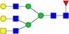	-	-
14	1996.724	-	-	0.018	-	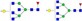	-	-
15	2012.719	-	0.032±0.021	0.078±0.026	0.048	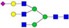	-	-
16	2028.714		0.054±0.015	0.041±0.006	-	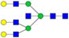	-	-
17	2067.687	0.043±0.007	0.045±0.007	0.083±0.005	0.044±0.003	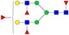	-	-
18	2101.755	-	0.053±0.038	-	-	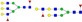	2047.713	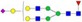
19	2174.772	0.036±0.020	0.199±0.072	0.216±0.089	-	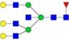	-	-
20	2215.798	-	-	0.021±0.007	-	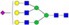	2157.756	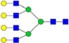
21	2231.793	-	0.020±0.016	0.026±0.018	-	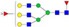	-	-
22	2247.813	-	0.015	-	-	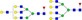	-	-
23	2320.829	-	0.051±0.038	-	-	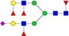	2262.788	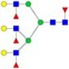
24	2377.851	-	0.010±0.001	0.038±0.003	-	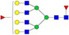	-	-
25	-	-	0.069	0.020	-		2319.809	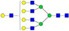
26	2393.846		0.036±0.0002			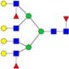		
27	2466.887	-	0.028	-	-	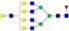	-	-
28	2539.904	-	0.214±0.077	0.147±0.048	-	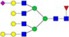	-	-
29	-	-	0.017±0.014	0.015±0.012	-		2538.908	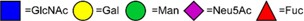
30	2612.945	-	0.009	-	-	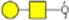	-	-
31	2685.962	-	0.036±0.027	-	-	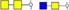	-	-
32	2758.978	-	0.006±0.001	-	-	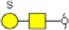	-	-
33	2832.02	-	0.013	-	-	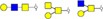	-	-
34	2905.036	-	0.011±0.003	0.020±0.006	-	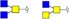	-	-
35	-	-	0.006	-	-		2993.052	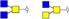

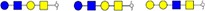

N-glycans with SD were identified in two independent experiments, and N-glycans without SD were identified in only one experiment.

Relative variation of major types of N-glycans in the four cell lines is summarized in Table 3. The percentage of high-mannose-type N-glycan structures was greater (93.0%) in GES-1 than in SGC-7901 (54.8%) or HGC-27 (70.5%). In contrast, complex-type N-glycan structures had a lower percentage in GES-1 (7.0%) than in SGC-7901 (45.2%) or HGC-27 (29.5%). Bi-, tri-, tetra-, and penta-antennary N-glycan structures (particularly triantennary) were suppressed in GES-1. Percentages of fucosylated complex-type N-glycan structures were higher in SGC-7901 (37.9%) and HGC-27 (21.3%) than in GES-1 (7.0%). The role of sialylated N-glycans in gastric cancer was investigated using acetohydrazide, which modifies both α2-3-linked and α2-6-linked sialic acids. Sialylated N-glycans were then annotated using the GlycoWorkbench program (Figure 3; sialic acid residues labeled as “Ah”).

**Table 3:** Relative variation of major types of N-glycans in the four cell lines

Glycan type	GES-1	SGC-7901	HGC-27	MGC-803
	(%)	(%)	(%)	(%)
High-mannose	93.0	54.8	70.5	95.8
Hybrid	0.0	1.3	0.0	1.1
Complex	7.0	45.2	29.5	3.1
Biantennary	5.2	30.8	11.5	3.1
Triantennary	1.8	16.2	22.7	0
Tetra-antennary	0	7.5	5.3	0
Sialylated	0	3.0	1.2	0
Fucosylated	7.0	37.9	21.3	3.2

### O-Glycan profiles obtained by MALDI-TOF-MS

**Figure 5:**
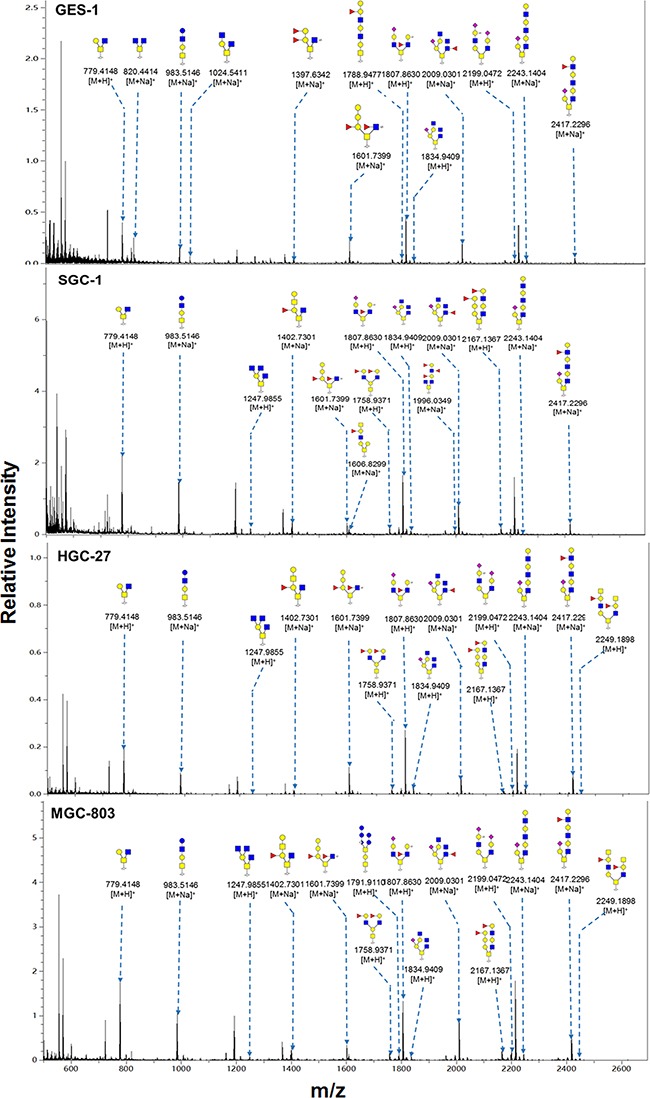
MALDI-TOF/TOF-MS spectra of total O-glycans in the four cell lines O-glycans were characterized in the four cell lines (GES-1, SGC-7901, HGC-27, and MGC-803 cells) by MALDI-TOF/TOF-MS as described in M&M. Representative spectra from triplicate experiments are shown. Detailed glycan structures were analyzed using the GlycoWorkbench program. Proposed structures and their m/z values are shown for each peak.

Representative MALDI-TOF-MS spectra of O-glycans with signal-to-noise ratios >4 from total glycoproteins were also annotated using GlycoWorkbench software program. A total of 36 O-glycan structures were identified in the four cell lines, 22 in GES-1, 21 in SGC-7901, 25 in HGC-27, and 29 in MGC-803 (Figure 5 and Table 4). A total of 12 O-glycans were presented in all the four cells. Two O-glycans with m/z 1247.986 ((GlcNAc)_3_(Gal)_1_(GalNAc)_1_) and 1606.830 ((Fuc)_1_(GlcNAc)_2_ (Gal)_3_(GalNAc)_1_) were specifically identified in SGC-7901, HGC-27, and MGC-803. One O-glycans with m/z 1614.859 ((GlcNAc)_1_(Gal)_4_(GalNAc)_2_) was specifically identified in GES-1. Interestingly, the relative intensity of Thomsen-Friedenreich antigen (TF antigen or T antigen) with m/z 512.307 (Gal(β1-3)GalNAc) was greater (18.69%) in GES-1 than in HGC-27 (5.53%) or MGC-803 (6.80%), and slightly higher in GES-1 than SGC-7901 (17.99%) (Table 4).

**Table 4:** Proposed structures and their molecular ions in MALDI spectra of O-linked glycans in the four cell lines

No.	Calculated m/z	Relative intensity				Glycan Structure	Type
		GES-1	SGC-7901	HGC-27	MGC-803		
1	512.307	0.1869±0.0011	0.1799±0.0014	0.0553±0.0001	0.0680±0.0002	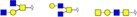	core 1
2	575.315		0.2947±0.0010			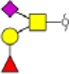	core 3,5
3	600.23	0.0813±0.0005		0.2438±0.0012	0.1167±0.0004	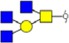	core 1
4	779.415	0.1401±0.0003	0.1502±0.0006			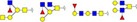	core 1,2,3
5	798.459				0.0351±0.0002	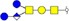	core 4,5
6	820.441	0.0717±0.0003		0.0396±0.0001	0.0641±0.0003	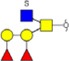	core 4,5
7	983.515	0.0789±0.0002	0.0848±0.0005	0.1347±0.0007	0.1031±0.0006	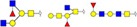	core 1,2,6
8	1024.541	0.0258		0.0308±0.0001	0.0325±0.0002	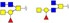	core 2,4,6
9	1069.551				0.0186±0.0001	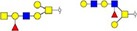	core 1
10	1247.986		0.0122	0.0309±0.0003	0.0203±0.0001	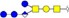	core 2
11	1380.748		0.0082±0.0001			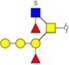	Core 1,2,3,4
12	1383.711			0.0102	0.0073	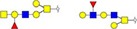	core 8
13	1397.634	0.0220±0.0001	0.0231±0.0001	0.0286±0.0001	0.0306±0.0001	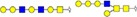	core 2
14	1402.73		0.0282±0.0002		0.0202±0.0001	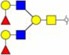	core 1,2,3
15	1421.775				0.0145±0.0001	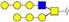	core 2,4
16	1584.848	0.0069		0.0105		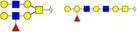	core 1,2
17	1587.811			0.0150±0.0001	0.0097	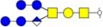	core 8
18	1601.74	0.1004±0.0002	0.0348±0.0002	0.1524±0.0005	0.0819±0.0003	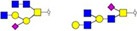	core 2
19	1606.83		0.0059	0.0118	0.0113	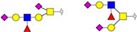	core 1,2
20	1614.859	0.0071				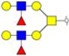	core 1,5
21	1758.937	0.0210±0.0001	0.0234±0.0001	0.0408±0.0001	0.0333±0.0001	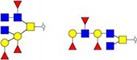	core 1
22	1777.932		0.0061			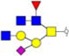	core 2
23	1788.948	0.0106	0.0108±0.0001	0.0209±0.0001	0.0099	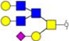	core 1
24	1791.911	0.0213±0.0001	0.0161±0.0001		0.0144±0.0001	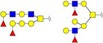	core 8
25	1834.941	0.0296±0.0001	0.0136±0.0001	0.0370±0.0001	0.0275±0.0001	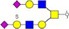	core 2,3
26	1857.969			0.0132		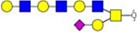	core 1,2
27	1963.037	0.0077	0.0127		0.0155	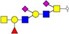	core 1
28	1996.035	0.0137±0.0001	0.0122±0.0001	0.0105	0.0143	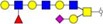	core 2,4
29	2009.03	0.1310±0.0006	0.1006±0.000	0.0752±0.0003	0.1404±0.0004	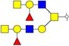	core 2
30	2039.041	0.0177±0.0001		0.0103	0.0140	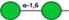	core 2
31	2167.137	0.0259±0.0002	0.0215±0.0001	0.0187±0.0001	0.0291±0.0001	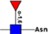	core 1,2
32	2199.047	0.0279±0.0001		0.0.45±0.0001	0.0208±0.0001	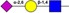	core 2
33	2243.14	0.0421±0.000	0.0177±0.0001	0.0233±0.0001	0.0298±0.0001	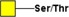	core 1
34	2370.204			0.0082	0.0076	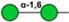	core 3
35	2417.23	0.0841±0.0007	0.0434±0.0001	0.0648±0.0003	0.0764±0.0002	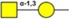	core 2
36	2249.16			0.0094	0.0085	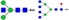	core 2

### Validation of differential glycopatterns in clinical gastric cancer tissues by lectin histochemistry

**Figure 6:**
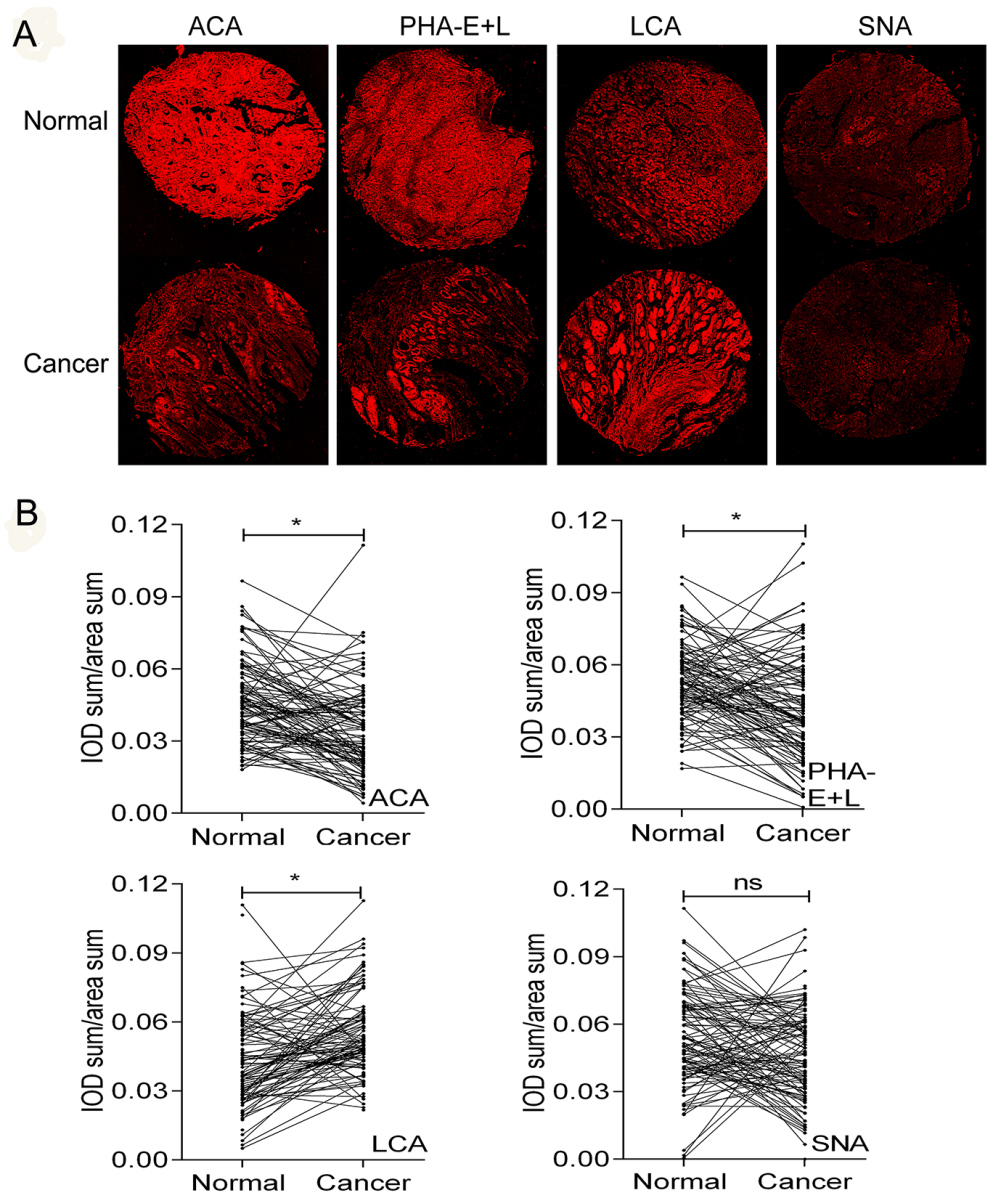
Differential glycopatterns in gastric cancer and normal gastric epithelial tissues evaluated by lectin histochemistry Four lectins (ACA, LCA, PHA-E+L, SNA) were used. Lectin histochemistry was performed in duplicate, and representative photos are shown (A and B). Normal: normal adjacent tissues. Cancer: gastric cancer tissues. IOD: integrated optical density Objective magnification: 10x. Scale bars: 200 μm.

Expression levels of LCA, SNA, ACA, and PHA-E+L in gastric cancer cells were significantly different from those in normal gastric epithelial cells. We examined the expression of the glycans recognized by these four lectins in clinical gastric cancer tissues and adjacent non-cancerous tissues (Figure 6A). Aberrant expression of the glycans recognized by LCA, PHA-E+L, and ACA was consistent with findings for gastric cancer cell lines (Figure 6B). In contrast, binding of SNA to clinical gastric cancer tissues vs. adjacent non-cancerous tissues was not significantly different (Figure 6B).

**Figure 7:**
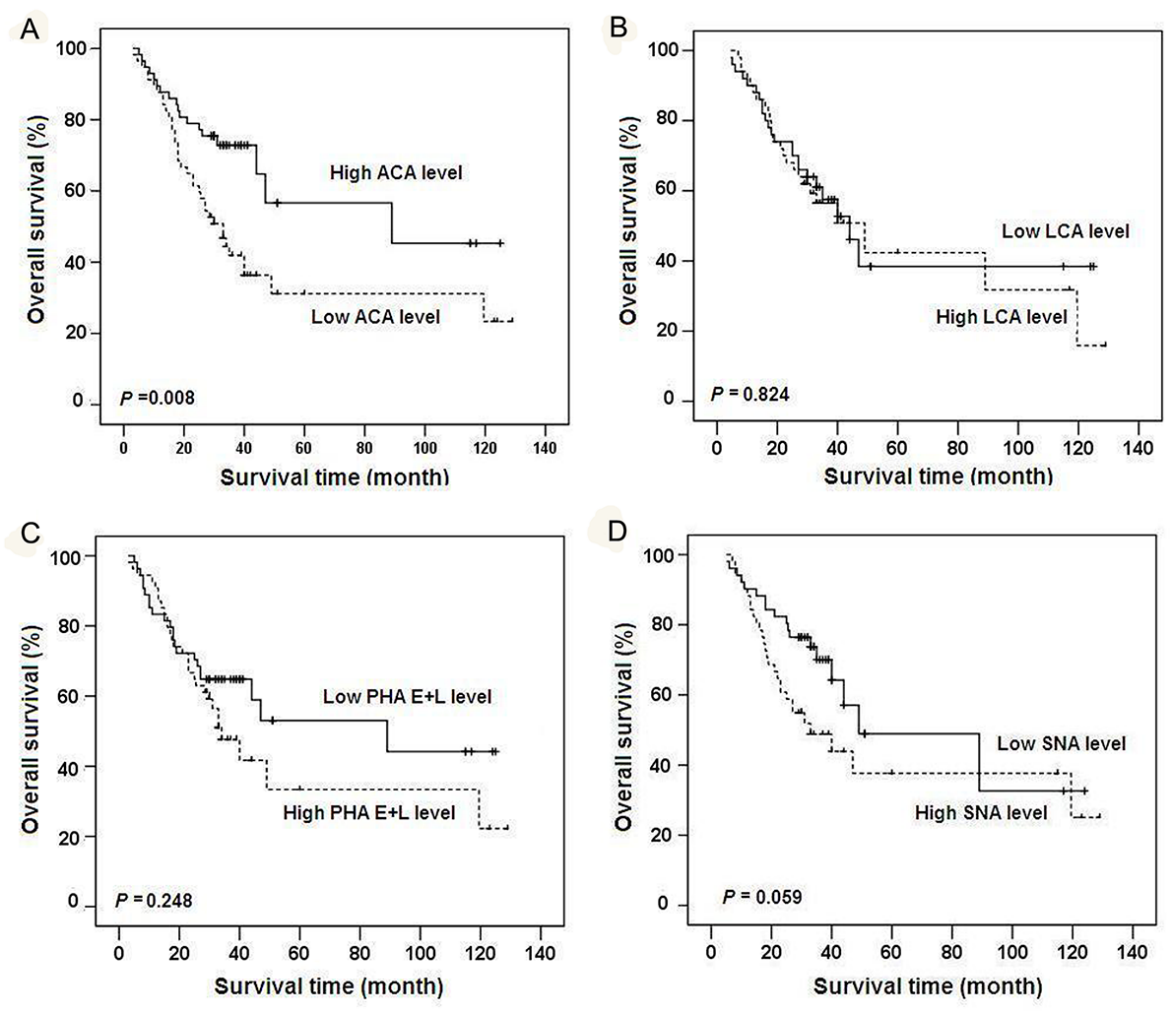
Kaplan-Meier overall survival curves for gastric cancer patients classified according to relative expression levels of four lectins Patients were classified into “relatively high” and “relatively low” groups using the median expression level of ACA A., LCA B., PHA-E+L C., or SNA D. in gastric cancer tissues as respective cutoff value.

To further evaluate the potential clinical value of these glycan structures in gastric cancer prognosis, patients were classified into “relatively high” and “relatively low” groups using the median level of each lectin in tumor tissues as a cutoff value. Notably, reduced ACA binding in gastric cancer was significantly correlated with higher TNM stage (*r*= -0.204, *P*= 0.026) and poorer prognosis (*P*= 0.008) (Figure 7). After adjusting for age, gender, T stage, N stage, TNM stage and tumor grade, Cox multivariate analyses showed that ACA expression is an independent prognostic factor for gastric cancer (HR=0.355; 95% CI= 0.198-0.636, *P*= 0.001). No significant associations were observed between binding capacities of the other three lectins (LCA, SNA, PHA-E+L) and clinicopathologic features of gastric cancer.

## DISCUSSION

Altered (aberrant) expression of N-glycans, O-glycans, and other glycoconjugates is often associated with tumor development and progression [19–21]. Gastric cancer is the most common type of epithelial cancer worldwide; however, the functional roles of glycosylation in gastric cancer remain mostly unclear. Better understanding of these functional roles requires more sophisticated and comprehensive analytical techniques. In this study, we integrated By integrating lectin microarray analysis with mass spectrometry, we were able to globally profile glycan expression in gastric cancer cells, and validate aberrant glycan expression in clinical gastric cancer tissues using specific lectins.

Lectin-based methodology is capable of revealing glycan structures of all glycoconjugates, *i.e*., glycoproteins, glycolipids, and glycosaminoglycans [22]. There are three effective lectin-based methods: (i) Simultaneous quantitative analysis of N-glycans and O-glycans using lectin microarrays with intact biological structures, following minimal sample preparation without release or derivatization of glycans [23, 24]. (ii) Lectin staining for identification and localization of glycoproteins and glycolipids in cells and tissues, and detection of subtle alterations in cellular glycosylation [25]. (iii) Lectin histochemistry, which is easily performed in a variety of cells, frozen or cryostat sections, and formalin-fixed or paraffin-embedded surgical specimens [26]. Combined application of these three methods ensures identification of correct glycosylation status in gastric cells or clinical samples. Using lectin microarray analysis, Huang et al. showed that VVA and MPL can be used as biomarkers to distinguish gastric cancer from gastric ulcer[12].

MS is a rapid and sensitive technique to detect glycan components and glycome profile of a biological sample [27]. The integrated strategy used in the present study and validation in both cell lines and clinical samples, led to several important findings. Gastric cancer cells showed clearly increased expression of core-fucosylated N-glycans, GalNAcα-Ser/Thr (Tn antigen), and Sia2-6Galβ1-4GlcNAc N-glycans, but decreased expression of biantennary N-glycans, Galβ1-3GalNAcα-Ser/Thr (T antigen), and (GlcNAc)_n_ N-glycans (Table 1).

Lectin microarray, lectin staining, and lectin histochemical analyses of gastric cancer cells and clinical samples revealed differential expression of core-fucosylation, biantennary N-glycans, and T antigen (recognized by LCA, PHA-E+L, and ACA, respectively), suggesting that these glycans are involved in development and progression of gastric cancer. There is increasing evidence for a role of core-fucose residue in a variety of physiological and pathological processes. Levels of core-fucosylated N-glycans (recognized by LCA) in certain serum proteins are undetectable in normal cells, but are higher in cancer cells [28–30]. On the other hand, Chen et al. observed lower total abundance of core-fucosylated residues in sera of gastric cancer patients [31]. In the present study, levels of core-fucosylated N-glycans were significantly higher in gastric cancer cells than in normal epithelial cells. Core-fucosylation, which is catalyzed by α1,6-fucosyltransferase (Fut8) in mammalian tissues, plays multiple roles in cancer. Increased core-fucosylation has been reported in prostate, ovarian, pancreatic, and non-small cell lung cancers [32]. High levels of core-fucose were also observed during the process of epithelial-mesenchymal transition [32]. Depletion of core-fucose in human IgG1 enhances antibody-dependent cell-mediated cytotoxicity during cancer therapy [33]. Disruption of the Fut8 gene in mice resulted in growth retardation, death during postnatal development, and lung emphysema [34].

In a study by Shimada et al., cancer patients and normal subjects showed similar levels of total free sialic acids [35]. However, levels of most sialylated oligosaccharides, and of α2-3 (but not α2-6) sialic acid that binds to lactose, were higher in advanced cancer patients than in normal subjects [35, 36]. In the present study, α2-6 sialic acid (recognized by SNA) was higher in gastric cancer cells than in normal cells, but no significant difference was observed between clinical gastric cancer samples and corresponding normal tissues. Larger sample sizes of gastric cancer at various stages will be required to clarify this point.

A high concentration of Tn antigen on the surface of cancer cells is often associated with aggressive or highly malignant properties. T antigen is formed by addition of one Gal residue to GalNAc to yield Gal(β1-3)GalNAc. We observed lower levels of T antigen (recognized by ACA) in gastric cancer cells correspondence the results from lectin array and O-glycan/N-glycan analysis, in contrast to previous reports of high T antigen levels in sera of gastric cancer patients [37]. A possible explanation of high Tn antigen level but low T antigen level in gastric cancer cells is the decreased enzymatic activity of β1,3-galactosyltransferase, which adds Gal to Tn substrate to form T antigen. Our survival analysis and multivariate analyses showed that prognosis was significantly worse for patients with low (vs. high) ACA binding capacity, suggesting that glycans recognized by ACA are potential prognostic factors for gastric cancer.

PHA-E+L includes PHA-E and PHA-L, which recognize bisecting GlcNAc and biantennary N-glycans, respectively. Overexpression of bisecting GlcNAc inhibited tumor progression in mice, perhaps through an effect on growth factor signaling [38]. Our lectin microarray analysis revealed no significant changes in bisecting GlcNAc, and reduced expression of biantennary N-glycans was observed in gastric cancer cells and clinical cancer tissue samples. We consider biantennary N-glycan as the novel identified N-glycan in this study.

Taken together, our findings from lectin microarray analysis, MS, and histochemical staining demonstrate increased expression of Tn antigen, N-glycan structures, and core-fucosylated N-glycans, and reduced expression of T antigen and biantennary N-glycans in gastric cancer cells and clinical samples. Our follow-up studies, in progress, are focused on alterations of these glycans and their related genes at the molecular level, the glycoproteins to which the altered glycans bind, and the roles of these glycans in functional modulation of the glycoproteins in gastric cancer.

## MATERIALS AND METHODS

### Cell lines and clinical samples

Gastric cancer cell lines SGC-7901 (metastatic stomach adenocarcinoma derived cell), HGC-27 (undifferentiated stomach adenocarcinoma cell), and MGC-803 (poorly differentiated stomach mucinous adenocarcinoma cell), and immortalized normal gastric epithelial cell line GES-1, were obtained from the Cell Bank of the Chinese Academy of Sciences (Shanghai). All cells were cultured in RPMI 1640 (HyClone; Logan, UT, USA) containing 10% fetal bovine serum (Gibco; Carlsbad, CA, USA) and 1× penicillin/ streptomycin (Gibco) at 37°C in 5% CO_2_ atmosphere.

Tissue microarrays including 120 pairs of gastric tissues and adjacent normal tissues (Supplementary Table S1) were supplied by the Affiliated Hospital of Jiangnan University. All patient materials were obtained with written informed consent from all subjects. This study and all the experimental protocols were approved by the Clinical Research Ethics Committee of Affiliated Hospital of Jiangnan University. All the study methods were consistent with the guidelines approved by the Committee.

### Total protein extraction

Cells (at 80-90% confluence) were lysed with Tissue Protein Extraction Reagent (T-PER) (Thermo Scientific; San Jose, CA, USA) according to the manufacturer's instructions. Protein content was determined by BCA assay (Beyotime; Shanghai).

### Lectin microarray analysis

Lectin microarray analysis was performed as described previously [39–41]. In brief, 37 commercial lectins from Vector Laboratories (Burlingame, CA, USA), Sigma-Aldrich (St Louis, MO, USA), and Calbiochem Merck (Darmstadt, Germany) were immobilized on a solid support at high spatial density. Glycoprotein samples labeled with fluorescent dye Cy3 (GE Healthcare; Buckinghamshire, UK) were applied and scanned with a GenePix 4000B confocal scanner (Axon Instruments; Union City, CA, USA). Raw values less than the mean background value were omitted from analysis. The median for each lectin was globally normalized to the sum of medians of all valid data for the 37 lectins. Each sample was repeated tested in three lectin microarrays and the mean value of each lectin from 3 repeated arrays and its SD were calculated.

### Lectin staining

Cells were cultured in 24-well plates with sterilized glycergel (DakoCytomation; Carpinteria, CA, USA) to 60-70% confluence, and stained with Cy3-labeled lectins (ACA, PHA-E+L, LCA, SNA) as described previously^14^.

### Separation of N-glycans, and amidation of sialylated N-glycans

Proteins (2 mg) from each cell line were concentrated and desalted using a size-exclusion spin ultrafiltration unit (Amicon Ultra-0.5 10 KD, Millipore; Billerica, MA, USA) as described previously [39, 42, 43]. In brief, proteins were denatured with 8 M urea, 10 mM DTT, and 20 mM IAM (Sigma-Aldrich), and centrifuged. Samples were washed with 50 mM NH_4_HCO_3_, and desalted by washing with deionized water. The desalted proteins were redissolved with 100 μL of 1M acetohydrazide (Sigma-Aldrich), 20 μL of 1 M HCl, and 20 μL of 2 M EDC (1-ethyl-3-(3-dimethyllaminopropyl) carbodiimide hydrochloride) (Sigma-Aldrich). The mixture was incubated at room temperature for 4 h to amidate sialylated N-glycans. The sample was further digested with PNGase F (New England BioLabs; Ipswich, MA, USA) (1:1000) overnight at 37°C. Released amidated N-glycans were collected and lyophilized.

### Desalting of N-glycans

Desalting was performed using Sepharose 4B (Sigma-Aldrich) as described previously [44]. Sepharose 4B in a microtube was washed with methanol/ H_2_O (1:1 v/v) (MW) and 1-butanol/ methanol/ H_2_O (5:1:1 v/v/v) (BMW) under centrifugation. Glycans were dissolved in 500 μL BMW and added to the microtube. The mixture was gently shaken for 45 min and washed three times with BMW. N-glycans were eluted with MW, collected, and lyophilized.

### Separation and permethylated derivatization of O-linked glycans

Glycoproteins were subjected to modify β-elimination protocol. The proteins were denatured and desalted using a size-exclusion spin ultrafiltration unit. 300 μL ammonia- borane complex, prepared in 28% ammonium hydroxide were added to samples and incubated for 24 h at 37°C and subsequently cool to room temperature prior to the addition of sufficient 1 M HCl to neutralize any residual ammonia - borane complex and ammonium hydroxide. This step was conducted in an ice bath. The samples were lyophilized, methanol-washed, and then dried three times to remove residual borate [45]. The glycans were cleaned-up using carbon SPE column (Grace Davison Discovery Sciences, USA) and then lyophilized. The resulting reduced glycans were subsequently subjected to permethylated derivatization. Four NaOH pellets (approximately 500 mg) were crushed in 10 mL anhydrous DMSO; 200 μL of this slurry and 50μL CH_3_I were then added to the dried glycans and the mixture was shaken vigorously for 20-30 mins. The mixture was extracted five times sequentially with a mixture of 2 mL water and 1 mL chloroform. Finally, the combined chloroform phases were dried under nitrogen. The permethylated O-glycans were further purified through carbon SPE column [46].

### Mass spectrometry

N-glycans were characterized by MALDI-TOF/TOF-MS (UltrafleXtreme, Bruker Daltonics; Bremen, Germany). Lyophilized N-glycans were resuspended in 5 μL MW, and 1 μL of the mixture was spotted onto an MTP AnchorChip sample target and air-dried. One μL of 20 mg/mL 2,5-dihydroxybenzoic acid (DHB) in MW was spotted to recrystallize the glycans. Mass calibration was performed using peptide calibration standards (250 calibration points; Bruker). Measurements were taken in positive-ion mode and the intense ions from MS spectra were subsequently selected and subjected to MS/MS. Representative MS spectra of N-glycans with signal-to-noise ratios >3 were chose and annotated using the GlycoWorkbench program with the accuracy < 1.0 (http://code.google.com/p/glycoworkbench/). Relative intensities were analyzed and generated using the FlexAnalysis software program (Bruker Daltonics). Relative variation was calculated by dividing the relative intensity of a particular type of N-glycan by the sum of N-glycan relative intensity in one scan, as described previously [47].

### Lectin histochemistry

Lectin histochemistry staining was performed on 4-mm sections of paraffin-embedded tissue samples for detection of lectin expression levels, as described previously^14^. Representative area and fluorescence intensity of tissue dots were measured using the Image-Pro Plus 6.0 software program (Media Cybernetics; Carlsbad, CA, USA) [48, 49].

### Statistical analyses

The quantitative or qualitative data was subjected to the Mann–Whitney U test or χ^2^ test, respectively. Spearman correlation analysis was performed to evaluate potential relationship between different variables. The survival curves were plotted according to the Kaplan–Meier method and verified by the log-rank test. Cox proportional hazards regression analysis was used to estimate the HRs and the 95% confidence intervals (CI). A value of *P* < 0.05 was considered to be statistically significant.

## SUPPLEMENTARY MATERIALS FIGURES AND TABLES




