# Supplementary material for: Identification of aberrantly expressed glycans in gastric cancer by integrated lectin microarray and mass spectrometric analyses
Source: Oncotarget. 2016 Nov 24;7(52):87284–300. doi: 10.18632/oncotarget.13539 (PMC5349988; doi:10.18632/oncotarget.13539)
Supplement: Supplementary file 1 [file oncotarget-07-87284-s001.pdf]

Identification of aberrantly expressed glycans in gastric cancer by integrated lectin microarray and mass spectrometric analyses

SUPPLEMENTARY FIGURE AND TABLES

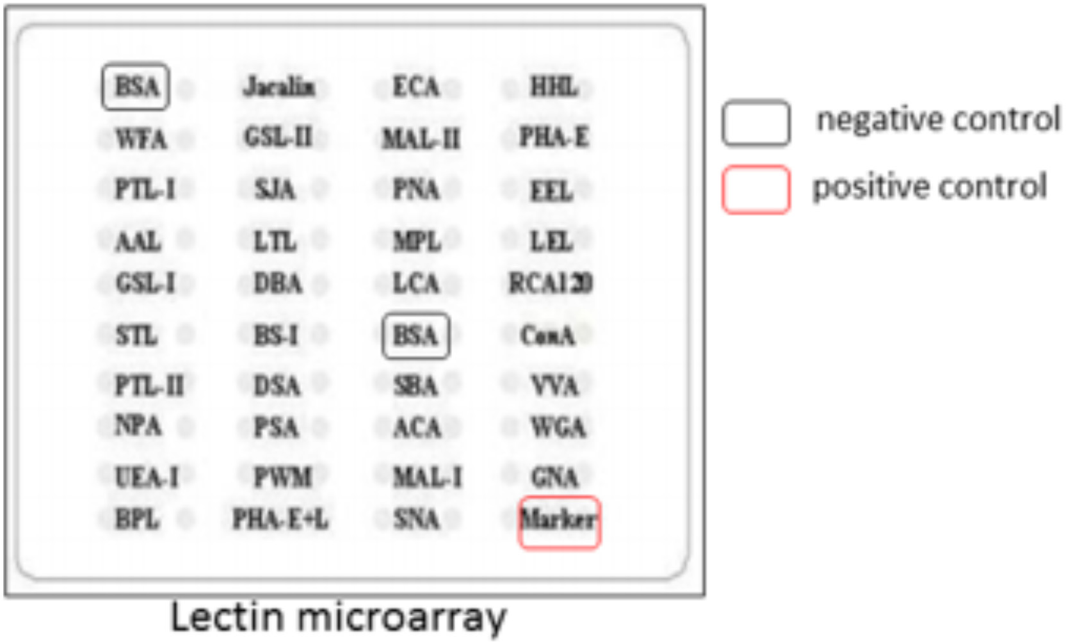

Supplementary Figure S1: lectin microarrays containing 37 lectins (Supplementary Table S2), two negative controls (BSA), and one positive control (Cy3-BSA).

Supplementary Table S1: Patients' information

Characteristics	gastric cancer (n=120)
Gender	
Male	94
Female	26
Age at diagnosis	
≥64	61
<64	59
Nodal status	
Positive	88
Negative	32
T stage	
T1-T2	26
T3-T4	94
Differentiation	
Well and moderate	60
Poor	60
TNM stage	
I-II	59
III-IV	61

Supplementary Table S2: Thirty-seven lectins and their relative intensity in gastric cancer and GSE-1 cell lines detected by lectin microarray.

See Supplementary File 1
