# Supplementary material for: Identification of aberrantly expressed glycans in gastric cancer by integrated lectin microarray and mass spectrometric analyses
Source: Oncotarget. 2016 Nov 24;7(52):87284–300. doi: 10.18632/oncotarget.13539 (PMC5349988; doi:10.18632/oncotarget.13539)
Supplement: Supplementary file 2 [file oncotarget-07-87284-s002.docx]

**Table S2. Thirty-seven lectins and their relative intensity in gastric cancer and GSE-1 cell lines detected by lectin microarray**

| **No.** | **Lectin** | **Binding structure** | MGC-803 /GSE-1 | HGC-27 /GES-1 | SGC-7901 /GSE-1 |
| --- | --- | --- | --- | --- | --- |
| 1 | Jacalin | Galβ1-3GalNAcα-Ser/Thr(T)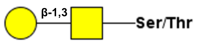  GalNAcα-Ser/Thr(T)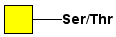 | 0.992 | 1.143 | 1.183 |
| 2 | ECA | Galβ-1,4GlcNAc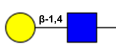 | 0.830 | 1.135 | 1.292 |
| 3 | HHL | Non-substituted α-1,6 Man 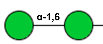 | 0.441 | 1.010 | 0.355 |
| 4 | WFA | GalNAcα/β1-3/6Gal 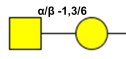 | 0.607 | 0.978 | 0.463 |
| 5 | GSL-II | GlcNAc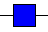  Galactosylated N-glycans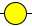 | 0.750 | 1.199 | 0.944 |
| 6 | MAL-II | Siaα2-3Galβ1-4Glc(NAc)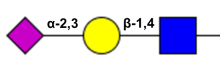 | 0.801 | 1.174 | 1.232 |
| 7 | PHA-E | Bisecting GlcNAc and Biantennary N-glycans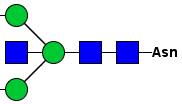 | 0.707 | 0.961 | 1.014 |
| 8 | PTL-I | αGalNAc and Gal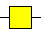, 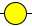 | 0.975 | 1.300 | 0.856 |
| 9 | SJA | Terminal GalNAc and Gal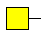,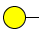 | 0.711 | 0.993 | 0.917 |
| 10 | PNA | Galβ1-3GalNAcα-Ser/Thr(T)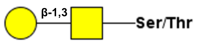 | 0.923 | 1.044 | 1.002 |
| 11 | EEL | Galα1-3(Fucα1-2)Gal 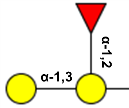 | 0.889 | 0.758 | 1.060 |
| 12 | AAL | Terminal Fucα-1,6GlcNAc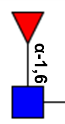  Fucα-1,3Galβ-1,4GlcNAc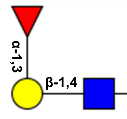 | 0.966 | 0.758 | 0.789 |
| 13 | LTL | sLe^x^, Le^x^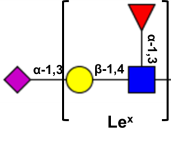  Fucα-1,3GlcNAc (core) 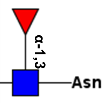； | 0.684 | 0.791 | 0.610 |
| 14 | MPL | αGalNAc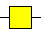 | 1.041 | 1.144 | 1.207 |
| 15 | LEL | Poly-LacNAc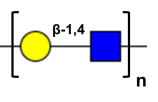  (GlcNAc)_n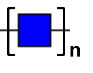_ | 1.235 | 1.396 | 0.865 |
| 16 | GSL-I | αGalNAc, αGal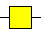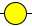  GalNAcα-Ser/Thr(Tn) 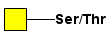 | 0.747 | 0.898 | 0.947 |
| 17 | DBA | GalNAcα-Ser/Thr(Tn) 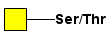  GalNAcα1-3Gal 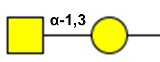 | 0.943 | 0.857 | 0.680 |
| 18 | LCA | Fucα-1,6GlcNAc (core)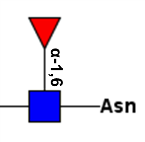 | 1.279 | 1.066 | 1.078 |
| 19 | RCA120 | Gal, GalNAc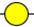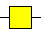 | 1.579 | 1.599 | 1.506 |
| 20 | STL | (GlcNAc)_n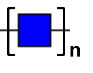_ | 1.623 | 0.949 | 1.170 |
| 21 | BS-I | α-Gal, α-GalNAc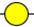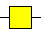 | 1.100 | 0.953 | 1.154 |
| 22 | ConA | Branched and terminal Man,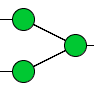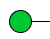  terminalGlcNAc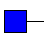 | 1.176 | 0.966 | 1.027 |
| 23 | PTL-II | Gal 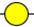 | 1.162 | 0.759 | 1.145 |
| 24 | DSA | GlcNAc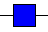 | 1.089 | 0.989 | 1.194 |
| 25 | SBA | Terminal GalNAc (especially GalNAcα1-3Gal)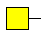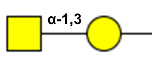 | 1.277 | 1.096 | 1.311 |
| 26 | VVA | GalNAc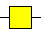  GalNAcα-Ser/Thr(Tn)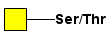 | 1.204 | 1.352 | 1.286 |
| 27 | NPA | Non-substituted α-1,6Man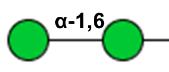 | 1.166 | 0.741 | 0.733 |
| 28 | PSA | Fucα-N-acetylchitobiose-Man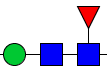 | 1.250 | 0.935 | 1.237 |
| 29 | ACA | Galβ1-3GalNAcα-Ser/Thr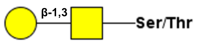 | 1.300 | 1.370 | 1.334 |
| 30 | WGA | Multivalent Sia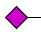； (GlcNAc)_n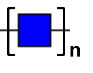_ | 1.182 | 0.813 | 1.154 |
| 31 | UEA-I | Fucα1-2Galβ1-4Glc(NAc)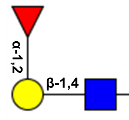 | 1.230 | 0.924 | 1.142 |
| 32 | PWM | GlcNAc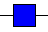 | 1.251 | 0.905 | 1.291 |
| 33 | MAL-I | Galβ-1,4GlcNAc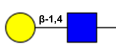 | 1.319 | 1.178 | 1.185 |
| 34 | GNA | Terminalα-1,3 Man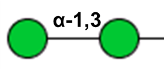 | 1.144 | 0.616 | 0.699 |
| 35 | BPL | Galβ1-3GalNAc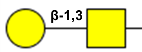 | 1.145 | 0.839 | 1.073 |
| 36 | PHA-E+L | Bisecting GlcNAc and biantennary N-glycans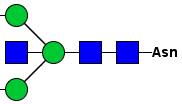  Tetra-antennary complex-type N-glycan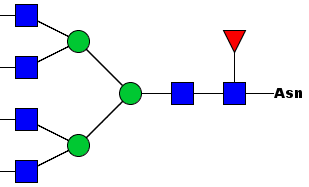 | 1.094 | 0.983 | 1.148 |
| 37 | SNA | Sia2-6Galβ1-4GlcNAc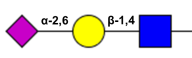 | 1.443 | 0.982 | 1.191 |
